# Supplementary material for: Intravenous Administration of Human-Derived Mesenchymal Stem Cell-Conditioned Medium for Patients with General Malaise
Source: J Clin Med. 2025 Aug 20;14(16):5884. doi: 10.3390/jcm14165884 (PMC12387221; doi:10.3390/jcm14165884)
Supplement: Supplementary file 1 [file jcm-14-05884-s001.zip › jcm-3776198-supplementary.pdf]

Supplementary

Table S1. Patient background.

Patient No.	Age (years)	Sex	Chief complaint	Past medical history
001	81	F	General malaise, respiratory distress	Suspicion of coronary angina pectoris
002	91	M	General malaise, back pain	Chronic cardiac insufficiency
003	82	M	General malaise	Suspicion of dementia
004	87	M	General malaise	Cataract
005	75	M	General malaise, throat discomfort	Myocardial infarction, cerebral infarction, gallbladder cancer
006	88	M	General malaise, lower body/right shoulder pain, stiff shoulders	Hypertension, heart failure, difficulty sweating, right eye blindness
007	74	F	General malaise, respiratory distress	Gallbladder removal, pancreatic cancer, ovarian tumor, hysterectomy, splenectomy, appendectomy
008	87	F	General malaise, hip joint pain	Hypertension, heart failure, stroke
009	81	F	No strength in body	Hypertension, papillary carcinoma
010	81	F	General malaise, insomnia	Hypertension, heart failure, diabetes mellitus, cerebral aneurysm
011	82	F	General malaise, back pain	Hypertension, hyperlipidemia, heart failure, ovarian cyst, cataracts, green cataracts
012	66	M	General malaise, back pain, no strength in body	Gallbladder removal, diabetes mellitus, cataracts
013	63	F	General malaise, shoulder and arm pain, no strength in body	Uterine fibroids, ileus
014	56	F	General malaise, tremor in the left hand, hair loss	Hypertension, hyperlipidemia
015	65	M	General malaise	Hyperlipidemia, headache
016	37	F	General malaise, muscle and joint pain	Hysterectomy (cervical cancer)
017	70	M	General malaise	Acute myocardial infarction, hypertension, diabetes mellitus
018	53	M	General malaise, pain in the lower back and limbs, heaviness in the chest	Diabetes, heart failure, hernia
019	72	M	General malaise, daytime sleepiness, post-COVID-19 symptoms	Hypertension, angina pectoris, diabetes mellitus

The background (age, sex, chief complaint, and past medical history) of the 19 participants interviewed in the pre-administration interview. M, male; F, female.

Table S2. Changes due to conditioned medium administration.

Patient No.	Blood collection	CRP (mg/dL）	STAS (ascorbic acid eq. (mM))	Adiponectin (μg/mL)	Self-reported symptom
001	Before 1st dose	0.026	0.333	12.3	
	After 1st dose	0.053	0.218	13.4	C
	After 2nd dose	0.052	0.420	14.2	C
	After 3rd dose	0.031	0.358	14.6	C
002	Before 1st dose	0.112	0.445	5.9	
	After 1st dose	0.321	0.556	6.2	N
	After 2nd dose	0.085	0.573	7.3	C
	After 3rd dose	0.107	0.747	7.9	C
003	Before 1st dose	0.043	0.295	25.1	
	After 1st dose	0.047	0.406	23.0	O
	After 2nd dose	0.370	0.318	27.6	O
	After 3rd dose	0.041	0.399	26.7	C
004	Before 1st dose	0.048	0.750	43.5	
	After 1st dose	0.056	0.961	58.1	N
	After 2nd dose	3.210	0.903	44.4	N
	After 3rd dose	0.132	0.832	58.6	O
005	Before 1st dose	0.071	0.576	27.5	
	After 1st dose	0.052	0.757	23.7	O
	After 2nd dose	0.083	0.449	25.9	O
	After 3rd dose	0.122	0.510	23.3	C
006	Before 1st dose	0.392	0.491	7.1	
	After 1st dose	0.016	0.764	4.9	C
	After 2nd dose	0.954	0.491	5.2	C
	After 3rd dose	0.110	0.401	8.7	N
007	Before 1st dose	0.031	1.183	12.8	
	After 1st dose	0.061	1.220	12.2	N
	After 2nd dose	0.042	1.092	12.2	C
	After 3rd dose	0.039	0.765	14.1	C
008	Before 1st dose	0.493	0.489	9.6	
	After 1st dose	0.492	1.283	9.9	C
	After 2nd dose	0.456	1.186	9.1	C
	After 3rd dose	0.293	1.289	8.7	C
009	Before 1st dose	0.091	0.744	14.2	
	After 1st dose	0.702	0.408	16.6	C
	After 2nd dose	0.042	0.535	16.9	N
	After 3rd dose	0.010	0.501	16.9	C
010	Before 1st dose	0.105	0.474	11.4	
	After 1st dose	0.107	1.075	11.0	N
	After 2nd dose	0.154	0.949	12.2	O
	After 3rd dose	0.078	0.291	12.5	N
011	Before 1st dose	0.042	0.679	16.2	
	After 1st dose	0.039	0.607	18.8	O
	After 2nd dose	0.019	0.504	15.0	N
	After 3rd dose	0.040	0.615	15.2	N
012	Before 1st dose	0.017	0.474	4.3	
	After 1st dose	0.021	1.075	3.6	N
	After 2nd dose	0.113	0.949	3.5	C
	After 3rd dose	0.047	0.291	4.1	O
013	Before 1st dose	0.030	0.420	13.9	
	After 1st dose	0.056	0.313	15.5	C
	After 2nd dose	0.021	0.300	10.9	N
	After 3rd dose	0.020	0.355	12.7	C
014	Before 1st dose	0.002	0.408	9.8	

	After 1st dose	0.007	0.000	7.8	C
	After 2nd dose	0.027	0.232	10.5	C
	After 3rd dose	0.009	0.339	10.6	C
015	Before 1st dose	0.060	0.510	4.7	
	After 1st dose	0.005	0.838	4.1	C
	After 2nd dose	0.023	0.446	6.5	C
	After 3rd dose	0.010	0.504	4.2	O
016	Before 1st dose	0.455	0.378	15.4	
	After 1st dose	0.036	0.000	15.9	C
	After 2nd dose	0.043	0.343	18.0	C
	After 3rd dose	0.031	0.330	16.5	O
017	Before 1st dose	0.026	0.421	4.7	
	After 1st dose	0.033	0.450	5.2	C
	After 2nd dose	0.041	0.306	9.7	O
	After 3rd dose	0.045	0.307	12.0	O
018	Before 1st dose	0.087	0.617	6.6	
	After 1st dose	0.059	0.568	8.8	N
	After 2nd dose	0.039	0.439	8.9	N
	After 3rd dose	0.054	0.641	14.0	N
019	Before 1st dose	0.275	0.384	8.3	
	After 1st dose	0.291	0.261	7.6	C
	After 2nd dose	0.324	0.432	9.4	C
	AAfter 3rd dose	0.083	0.418	7.2	C

The blood collection values, and changes in subjective symptoms were shown for 19 participants when the conditioned medium was administered thrice. In the interview after administration, if the main complaint was improved, a "C" was entered in the rightmost column; if an improvement other than the main complaint was observed, an "O" was entered; and if no change was observed, an "N" was entered. CRP, C-reactive protein; STAS, serum total antioxidant status.
